# Validation of a Combined Transcriptome and T Cell Receptor Alpha/Beta (TRA/TRB) Repertoire Assay at the Single Cell Level for Paucicellular Samples

**DOI:** 10.3389/fimmu.2020.01999

**Published:** 2020-08-28

**Authors:** Nicolle H. R. Litjens, Anton W. Langerak, Amy C. J. van der List, Mariska Klepper, Maaike de Bie, Zakia Azmani, Alexander T. den Dekker, Rutger W. W. Brouwer, Michiel G. H. Betjes, Wilfred F. J. Van IJcken

**Affiliations:** ^1^Department of Internal Medicine Section Nephrology and Transplantation, Erasmus MC, University Medical Center, Rotterdam, Netherlands; ^2^Laboratory Medical Immunology, Department of Immunology, Erasmus MC, University Medical Center, Rotterdam, Netherlands; ^3^Center for Biomics, Erasmus MC, University Medical Center, Rotterdam, Netherlands; ^4^Department of Cell Biology, Erasmus MC, University Medical Center, Rotterdam, Netherlands

**Keywords:** single cell transcriptomics, single T cell receptor repertoire analysis, cDNA, combined transcriptome and T cell receptor repertoire assay, low cell number

## Abstract

Transcriptomics can be combined with TRA and TRB clonotype analysis at the single cell level. The aim of this study was to validate this approach on the ICELL8 Single-Cell system and to evaluate its usefulness to analyse clinical paucicellular samples. For this purpose, we carefully selected T cell lines with defined TRA/TRB clonotypes as well as clinical samples enriched for CD3^+^ T cells that possess a complex TCR repertoire. Low cell numbers of the different samples were dispensed in a chip on the ICELL8 Single-Cell System. Two sequencing libraries were generated from each single cell cDNA preparation, one for the TRA/TRB repertoire and one for the 5′ ends of transcripts, and subsequently sequenced. Transcriptome analysis revealed that the cell lines on average express 2,268 unique genes/cell and T cells of clinical samples 770 unique genes/cell. The expected combined TRA/TRB clonotype was determined for on average 71% of the cells of the cell lines. In the clinical samples the TRA/TRB repertoire was more complex than those of the cell lines. Furthermore, the TRB clonotype distribution of the clinical samples was positively correlated to frequencies of TCRVβ families in CD3^+^ T cells obtained by a flow cytometry-based approach (Spearman's Rho correlation coefficient 0.81, *P* = 6.49 ^*^ 10^−7^). Combined analyses showed that transcriptome-based cell type-specific clusters in clinical samples corresponded to clinical features such as CMV status. In conclusion, we showed that the ICELL8 Single-Cell System enabled combined interrogation of both TRA/TRB repertoire and transcriptome of paucicellular clinical samples. This opens the way to study the response of single T cells within heterogeneous samples for both their transcriptome and TRA/TRB clonotypes in disease or upon treatment.

## Introduction

The human body contains a sophisticated immune system that distinguishes self from non-self-antigens. This requires an adaptive system that is able to cope with a great range of antigens. T cells recognize antigens by their unique T cell receptor (TCR), either of the TCRαβ or TCRγδ type. Approximately 95% of T cells express a TCRαβ receptor, consisting of a TCRα and a TCRβ chain. These TCR chains are highly diverse in their variable domains. In each single cell a unique TCR encoding gene is formed through a complex recombination process involving either V and J genes (TCRα or TRA locus) or V, D, and J genes (TCRβ or TRB locus) (1). This V(D)J recombination process generates a huge TR repertoire diversity, especially in the V(D)J junction or complementarity determining region 3 (CDR3), which allows recognition of a broad range of antigens. Estimates of the number of possible different TCRαβ receptors amount to 10^12^ (2, 3). TCRαβ^+^ cells, which are either CD4^+^ (T helper) or CD8^+^ (T cytotoxic), comprise of different subsets reflecting different maturation stages and functions. Antigen-inexperienced or naïve T cells have a broad, unselected TCR repertoire (4) compared to antigen-experienced or memory T cells that contain TCR repertoires mostly consisting of particular antigen-selected specificities.

Assays to determine TCR repertoire diversity so far mainly focused on TCRβ chain profiling, varying from DNA- (5) or RNA sequencing-based (6) TRB bulk assays to flow cytometry-based single cell TCRVβ approaches (7). One major drawback of the bulk sequencing approaches is the large number of cells required. Flow cytometry-based assays for analyzing TCRVβ repertoire diversity at the single cell level are limited to 24 different TCRVβ families that collectively cover only 70% of the normal human TCRVβ repertoire. In addition, no information on TCRα profiles and thus the actual composition of the total TCRαβ receptor within a sample can be obtained using either one of these approaches. Furthermore, it remains difficult to examine changes in TCRαβ repertoire diversity within a heterogeneous pool of T cells, or low-abundant population like antigen-specific T cells without purifying them first or acquiring large numbers of cells.

Over the past years, single cell transcriptomics has become a popular approach and allows to detect the heterogeneity in gene expression among individual cells and the discovery of small subpopulations (8). Recently, single cell transcriptomics has been combined with TR transcript sequencing. This combination provides gene expression and TCR repertoire information on a single cell resolution. Several platforms exist for single cell combined TCR repertoire and transcriptomics analysis, including 10xGenomics and more recently the ICELL8 Single-Cell System (9, 10). Single cell transcriptomics usually requires 5–10 K cells (11–13), but little is known about the possibilities using these single cell-based molecular tools for questioning clinically relevant paucicellular samples (9, 10).

The aim of this study was to evaluate combined single cell transcriptome and TRA/TRB repertoire analysis with respect to clinically relevant samples with low cell numbers on the ICELL8 Single-Cell System. To this end we validated several aspects of the combined transcriptome and TR repertoire assay, using T cell lines with defined TRA/TRB clonotypes. We also employed clinical samples of end-stage renal disease patients prior to kidney transplantation, and their donors, which had previously been characterized in detail for the TRB repertoire using different approaches.

## Results

### Characterization of Samples and Generation of Single Cell Transcriptome and TR Repertoire Data

**Figure 1:**
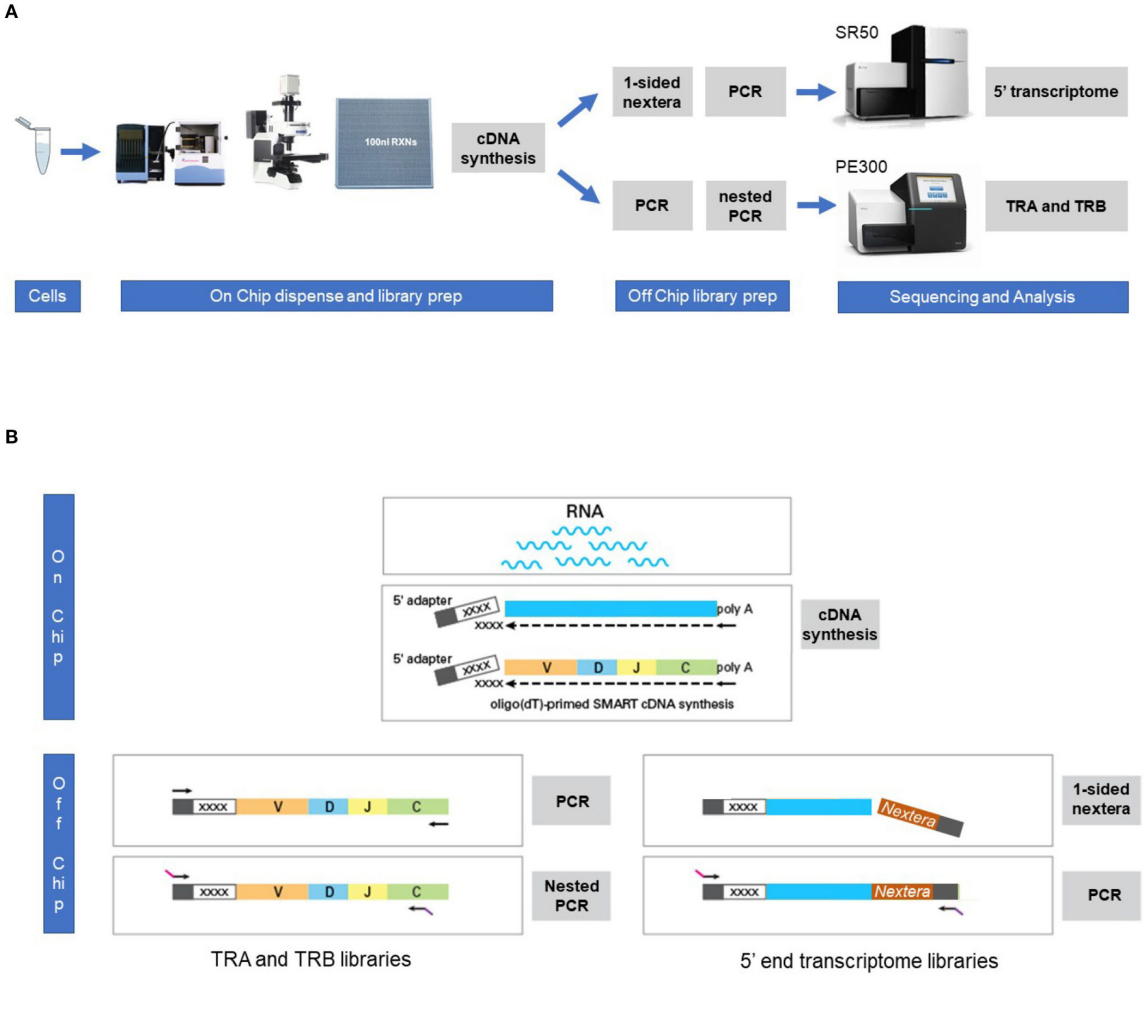
Workflow for the combined single cell transcriptome and TRA/TRB TCR repertoire assay on the ICELL8 Single-Cell System. **(A)** Single cells were stained and dispensed in an ICELL8 chip with 5184 nanowells by the ICELL8 dispenser. Wells with a single viable cell were selected using fluorescent microscopy. On chip cDNA was synthesized for the selected wells. The synthesized cDNA is collected by centrifugation and split in two aliquots. One aliquot was used to prepare a 5′ end transcriptome library, while the other aliquot was used to prepare libraries for TRA and TRB transcripts. The 5′ transcriptome library were sequenced single read 50 bp on an Illumina HiSeq2500 sequencer, whereas the TRA/TRB libraries were sequenced paired end 300 bp on an Illumina MiSeq system. Subsequent bioinformatic analysis yielded a correlated transcriptome profile and the TRA/TRB clonotypes for each single cell. **(B)** On chip messenger (m)RNA was isolated using an oligo(dT) primer. Copy DNA (cDNA) was synthesized for each single cell using a template switching oligo containing a well-specific barcode. cDNA of all wells was pooled by centrifugation and off-chip two sample preparations were performed, one for the TRA/TRB transcriptome and one for the 5′ ends of the transcripts. The TRA/TRB sequencing library was constructed by a first PCR making use of the fixed sequence in the switch oligo as well as constant region of the TRA/TRB locus and followed by a nested PCR. The 5′ end transcriptome library was prepared by cutting the 3′ end of by the nextera transposase and amplified by PCR making use of the fixed sequence of the switch oligo and tagged sequence by the transposase.

With the aim to evaluate the combined single cell transcriptome and TRA/TRB repertoire analysis in clinically relevant paucicellular samples, we implemented and applied a combined human TCR profiling and differential expression workflow on the ICELL8 Single-Cell System (Figure 1A). This high-throughput method employs a 5′RACE-like approach to determine both transcriptomic profiles and complete V(D)J variable regions of TR transcripts from individual cells (Figure 1B). Two cell lines, extensively characterized at the (c)DNA level with respect to TRA and TRB clonotypes, were used to validate the single cell approach with respect to TR profiling (Table 1). In addition, three clinical samples were chosen to evaluate the applicability of the combined assay for paucicellular samples (Table 1). The latter were characterized for their TRB repertoire diversity, using a multiplex DNA-based approach (15) as well as a flow cytometry-based approach with a kit containing antibodies directed against 24 different TCRVβ families (16).

**Table 1:** Characteristics of samples used for single-cell transcriptome analysis and TR.

**Sample name**	**Clinical characteristic**	**Sample type**	**Chip**	**TRA clonotype(s)**	**TRB clonotype(s)**	**References**
MOLT-17		T cell line (T-ALL type)	B	TRAV3-TRAJ5 TRAV12-1-TRAJ9^*^	TRBV20-1-TRBJ2-3 TRBV27-TRBJ1-1	(14)
HuT78		T cell line (CTCL type)	B	TRAV8-6-TRAJ37 TRAV20-TRAJ24	TRBV13-TRBJ1-2	(14)
Sample 1	CMV-seronegative	CD3+ enriched PBMC fraction	A,B	N.D.	skewed	(15, 16)
Sample 2	CMV-seropositive	CD3+ enriched PBMC fraction	A,B	N.D.	broad	(15, 16)
Sample 3	CMV-seropositive	CD3+ enriched PBMC fraction	A	N.D.	broad	(15, 16)

*CTCL, cutaneous T-cell lymphoma; CMV, cytomegalovirus; PBMC, peripheral blood mononuclear cells; TR, T-cell receptor repertoire; T-ALL, T-cell acute lymphoblastic leukemia; N.D., not determined*.

* *This TRA rearrangement has been documented in this cell line, but could not be traced back by single cell NGS or Sanger sequencing in the current DNA batch used*.

Peripheral blood mononuclear cells (PBMCs) from clinical samples were enriched for T cells in an untouched manner using Fluorescence Activated Cell Sorting (FACS). Cell viability and CD3^+^ T cell percentage were determined for the enriched fraction. Cell viability varied from 50 to 96% and samples contained >90% CD3^+^ T cells. To mimick paucicellular clinical samples, ~100–200 cells of these enriched fractions were processed on two independent chips to allow intra- and inter-chip comparisons. Transcriptome and TR sequencing results are summarized in Supplementary Table 1, Supplementary Figure 1. Overall, 43.4% (chip A) and 73.4% (chip B) of the cells dispensed yielded both transcriptome as well as TRA/TRB profile data. Notably, sample 3 had a lower success rate, which correlated with the lower viability of the cells in this particular sample (Supplementary Table 1), thereby underlining the importance of high cell viability for the combined transcriptome and TRA/TRB assay.

### Transcriptome Profiling

After filtering dead and doublet cells, 5′-specific transcriptome data were obtained from 55.7% (chip A) and 88.4% (chip B) of the cells dispensed (Supplementary Table 1, Supplementary Figure 1). Even though on average 62% of the dispensed cells of the clinical samples yielded transcriptome results vs. 93% of the cell lines (*P* = 0.01), the average percentage of mapped reads was not different between cell lines and clinical samples (89.8% of 31 K reads/cell vs. 90.1% of 38 K reads/cell, respectively). Furthermore, a clear correlation was observed between the number of detected genes and the number of reads (Supplementary Figure 2). On average 2,268 expressed genes per cell were detected for the cell lines, whereas in the clinical samples on average 770 expressed genes/cell could be identified. This difference is mostly likely explained by the cell lines being transcriptionally more active as they were cultured prior to use, whereas the clinical samples were processed immediately upon thawing. Notably, samples with lower cell viability expressed a lower number of genes, again stressing the importance of high cell viability of the starting material for combined single cell transcriptome and TRA/TRB analysis.

### Reproducibility of Transcriptome Profiles

**Figure 2:**
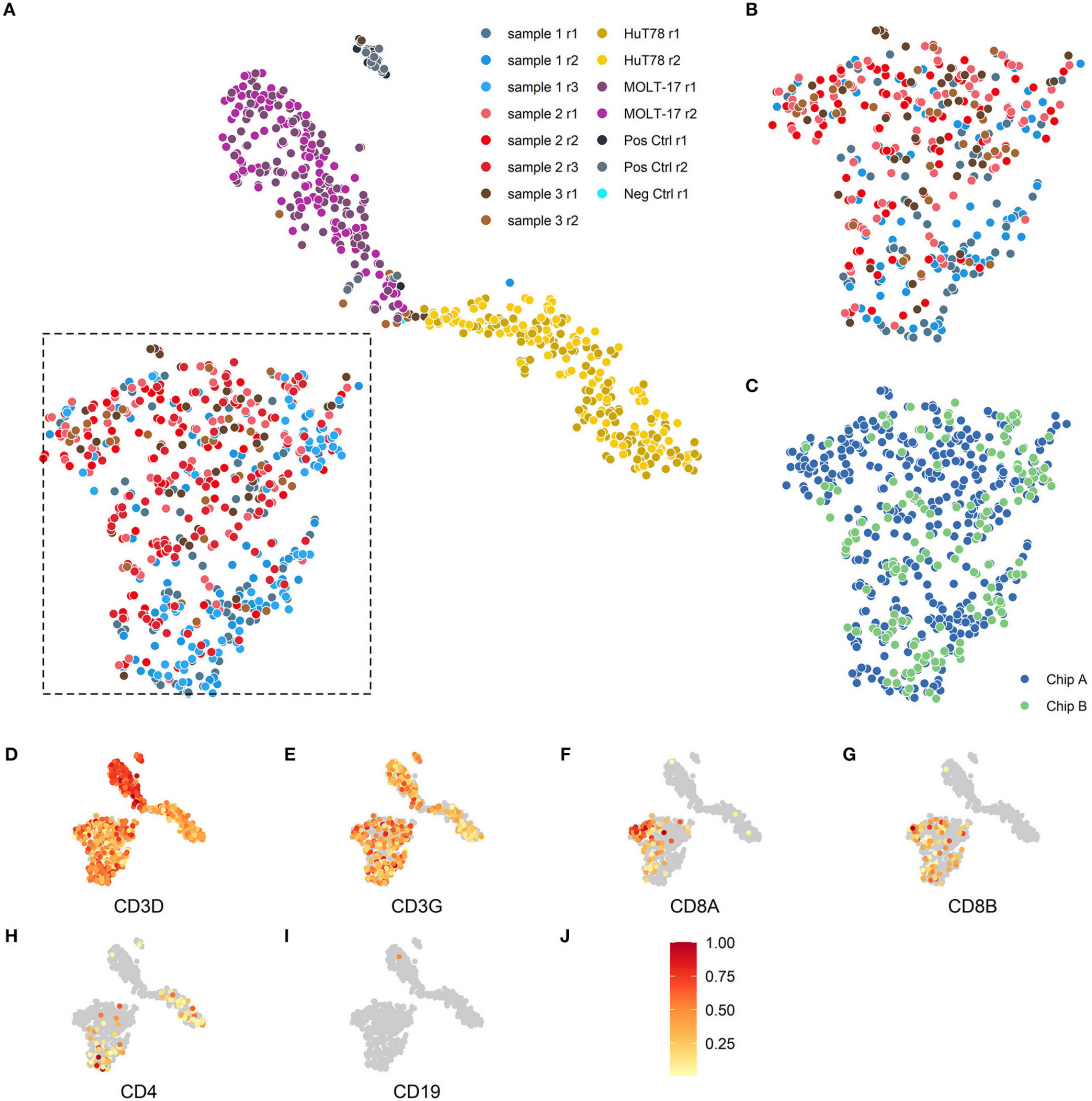
Two-dimensional t-SNE visualization based on cellular transcriptome profiles. Cells were projected on a two-dimensional plane based on their gene expression signals using the t-SNE method. **(A,B)** The cells are colored according to their respective samples and their replicates. **(A)** The cells of the 3 patient samples (blue, red, brown) are mainly placed together in the lower left section of the plot, the MOLT-17 cells (magenta) are placed in the top center section and the HuT78 cells (gold) are located in the right section of the plot. **(B)** Intra-chip reproducibility as shown by indicated section of the t-SNE projection including only cells from chip A. The replicates on chip A for the 3 patient samples show substantial overlap even though some sample specific preferences seem apparent. **(C)** Inter-chip reproducibility as shown by the overlap between the cells for chip A (blue) and chip B (green) in the indicated section of **(A)**. **(D–J)** The scaled gene expression **(J)** of the CD3 **(D,E)**, CD8 **(F,G)**, CD4 **(H)**, T-cell and CD19 **(I)** markers.

To evaluate the reproducibility of the single cell transcriptome data, cells were visualized via t*-*distributed stochastic neighbor embedding (t-SNE) (17) -based on the expression of the top 2,000 most variable genes. Separate clusters were detected for the two cell lines, one cluster for the clinical samples and one for the assay controls (Figure 2A). We next examined intra-chip reproducibility by comparing replicate samples (sample 1 r1 and 1 r2; sample 2 r1 and 2 r2; sample 3 r1 and 3 r2) that were dispensed and processed independently on the same chip (Figure 2B), whereas inter-chip reproducibility was examined by processing the same samples on two chips (i.e., sample 1 r1/1r2 and 2 r1/2r2 on chip A vs. 1r3 and 2 r3 on chip B, respectively) (Figure 2C). As replicate samples were all present in the same t-SNE cluster and no bias was detected in the locations of the replicate samples within the cluster, this indicated good intra- and inter-chip reproducibility.

### Cell Type Specificity Detection

As the clinical samples were enriched for T cells by depletion using a set of antibodies for non-T cell types (so-called “untouched” enrichment, see methods), we wanted to confirm that the cells are showing a typical T cell expression profile and checked for the presence of T cell (Figures 2D–H) and non-T cell markers (Figure 2I), respectively. Clear expression of *CD3D* (Figure 2D) and *CD3G* (Figure 2E) transcripts was detected in both clinical and cell line samples. T-cell lineage markers showed a differential expression profile for the clinical samples, indicating that these samples contained both *CD8A/CD8B*- (Figures 2F,G) as well as *CD4*–expressing T cells (Figure 2H); also the HuT78, but not the MOLT-17, cell line show *CD4* gene expression. Expression of the non-T cell *CD19* transcript (Figure 2I) was virtually absent in the clinical samples and the cell lines, which is in line with prior enrichment for T cells and their T cell clonal character, respectively.

### Single Cell TR Profiling

**Figure 3:**
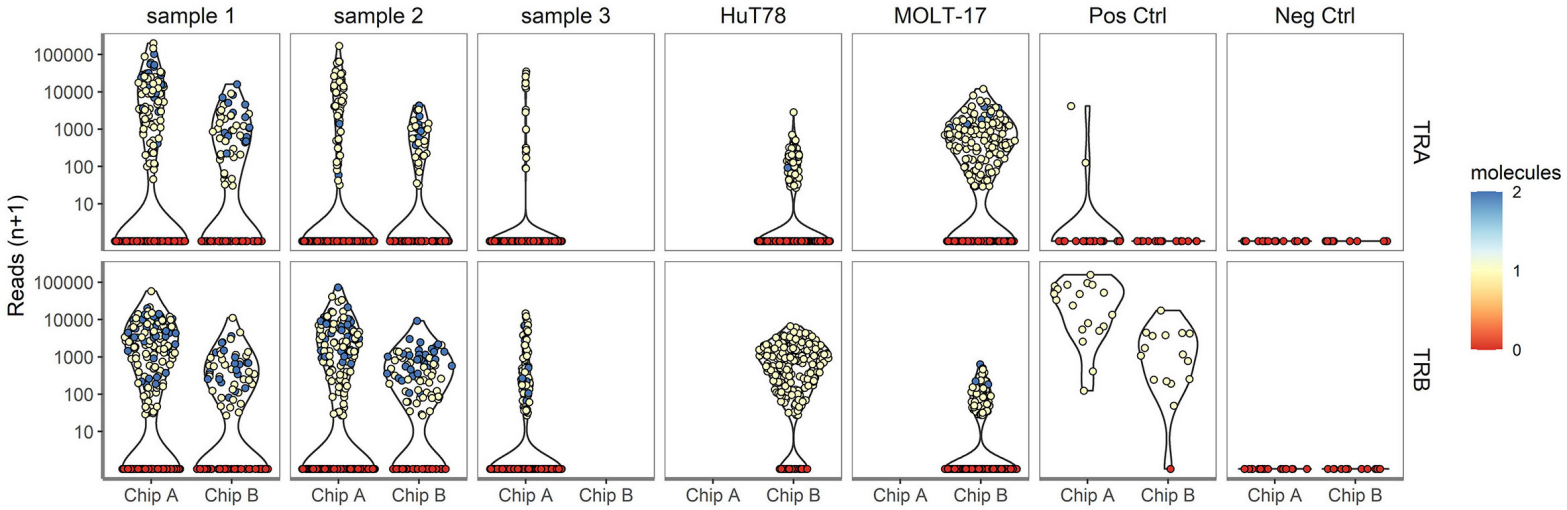
TRA/TRB clusters per single cell for different samples. The number reads for TRA (upper panel) and TRB (lower panel) clonotypes (depicted on the Y-axis) for each single cell plotted per sample for chip A and B (depicted on the X-axis).

Next, TR repertoire analysis was performed on the samples from chip A and B. The extensive PCR amplification required for the TR profiling gave rise to spurious clusters of clonotypes after sequencing. For this reason clonotypes were removed when (1) a clonotype was based on fewer than 25 reads; (2) <80% of the underlying alignments yielded the same protein sequence; (3) the clonotype comprised of <0% of the total alignments for that cell and/or 4) more than two clonotypes below the top 2 clonotypes per cell were detected. In the complexity analyses only the top V(D)J transcript was considered, even though two different TRA and two different TRB transcripts can theoretically be present per cell. Approximately, 49.9 and 75.7% of the cells dispensed in chip A and B, respectively, yielded a TCR profile (Supplementary Table 1, Supplementary Figure 1). A lower proportion of cells dispensed for the clinical samples tended to yield TRA/TRB clonotype results, as compared with the cell lines (averages of 56.1 vs. 77.1%, respectively; *P* = 0.07), indicating that TRA/TRB transcripts levels in the more quiescent cells of the clinical samples are relatively low. No difference was detected in the range of reads supporting either a TRA or TRB clonotype, indicating a roughly similar efficiency for TRA and TRB detection (Figure 3). In contrast to the transcriptome profiles, the cell lines did show differences in the number of TRA or TRB clusters, as compared with the clinical samples. Notably, actual TRA and TRB transcript levels differed between the cell lines; whereas HuT78 expressed higher TRB transcript levels, MOLT-17 cells more strongly expressed TRA transcripts (Figure 3).

### Validation of TRA/TRB Clonotypes in Cell Lines

**Figure 4:**
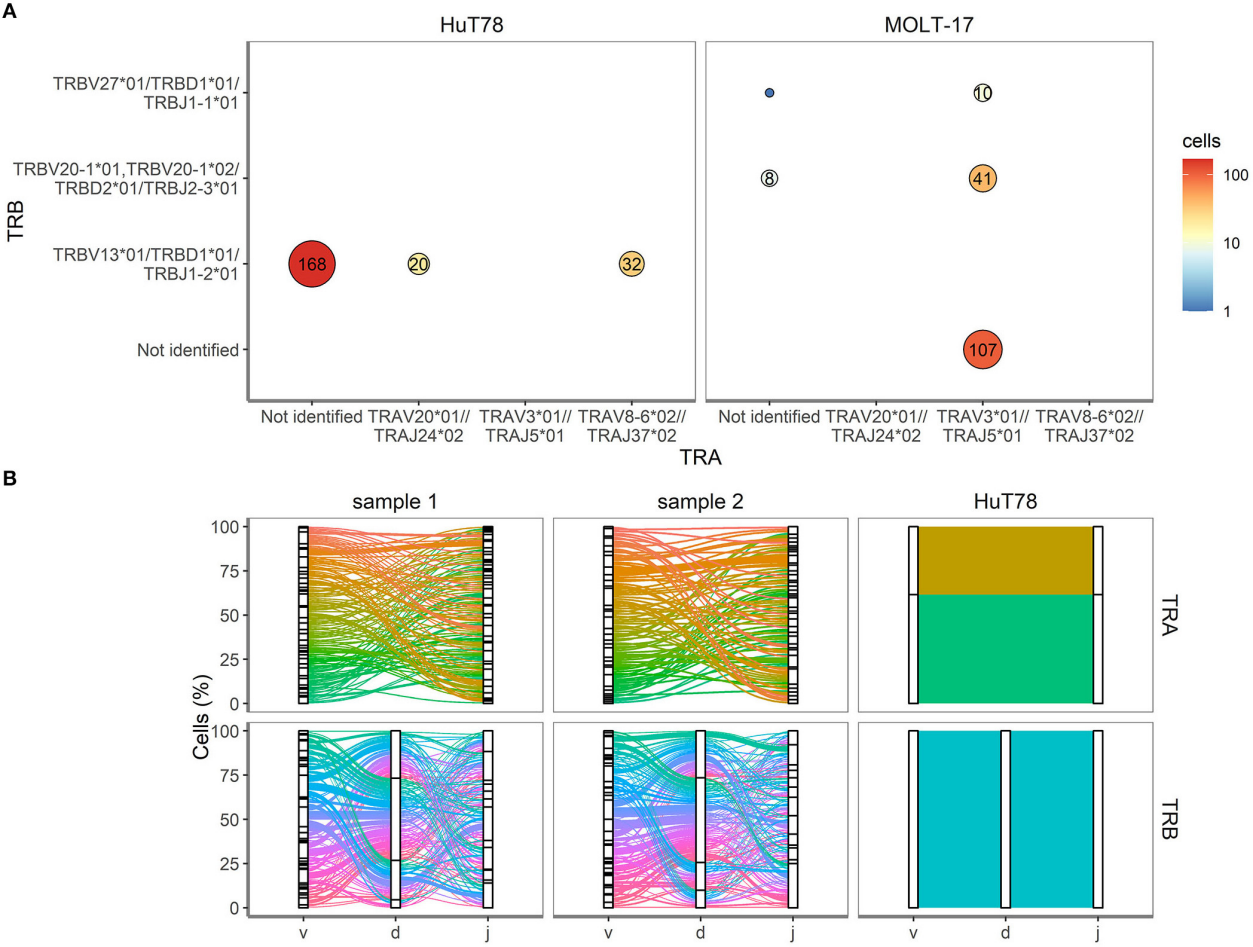
TRA and TRB clonotypes for cell lines and clinical samples. **(A)** V(D)J combinations for HuT78 and MOLT-17 cell lines and the mixture of the two cell lines. Size of the circle shows relative amount. Number inside circle shows number of single cells with specific clonotype. **(B)** Proportion of cells (Y-axis, %) is plotted against the V, D, J genes (X-axis) arranged as present in the human genome for TRA (top row) and TRB (bottom row). Colored lines differentiate unique V(D)J combinations, whereas the width of the line indicates the number of single cells with an identical V(D)J combination.

To validate the single cell approach for TR clonotype detection, the TR clonotypes of each individual single cell of the two T cell lines were determined based on the expressed TRA and TRB transcripts. For the MOLT-17 and HuT78 cell lines, predominant TR transcripts were apparent and expressed by almost every cell (Figure 4A). For HuT78, TRBV13^*^01-TRBD1^*^01-TRBJ1-2^*^01 transcripts were found either alone or in combination with TRAV20^*^01-TRAJ24^*^02 and TRAV8-6^*^01-TRAJ37^*^02 transcripts. In the MOLT-17 cell line, TRAV3^*^01-TRAJ5^*^01 clonotype was identified alone and in combination with TRBV20-01^*^01-TRBD2^*^01-TRBJ2-3^*^01 and TRBV27-01^*^01-TRBD1^*^01-TRBJ1-1^*^01 clonotypes. All identified clonotypes matched the expected TRA/TRB clonotypes obtained from Sanger and NGS data (14) (unpublished results) for each cell line. On average 63 and 80% of the dispensed HuT78 and MOLT-17 cells expressed the correct clonotypes, respectively (Table 2). The single cell TR repertoire assay enabled the detection of two novel, minor clonotypes: TRBV27^*^01/TRBD1^*^01/TRBJ1-1^*^01 and TRAV20^*^01//TRAJ24^*^02 clonotypes were detected in the MOLT-17and HuT78 cell lines, respectively (Figure 4A). Finally, one TRA clonotype (TRAV12.1-TRAJ9) that was previously identified in the MOLT-17 cell line, could not be traced in our single cell TR repertoire assay (Table 2). However, more detailed re-analysis using benchmark methods (Sanger, NGS) revealed that this clonotype was actually missing from the currently used cell line batch.

**Table 2:** Results TRA/TRB clonotypes for the two T cell lines.

**Cell line**	**DNA level**	**DNA OK?**	**Single cell**	**Sum score**	**Cells**	**Total cells**
MOLT-17	TRAV3-TRAJ5	Yes	TRAV3*01//TRAJ5*01	164,413	72	72
MOLT-17	TRAV12-1-TRAJ9	Yes	ND	NA	NA	72
MOLT-17	TRBV20-1-TRBJ2-3	Yes	TRBV20-1*01,TRBV20-1*02/TRBD2*01/TRBJ2-3*01	8,243	52	72
MOLT-17	TRBV27-TRBJ1-1	NA	TRBV27*01/TRBD1*01/TRBJ1-1*01	6,948	47	72
HuT78	TRAV8-6-TRAJ37	Yes	TRAV8-6*02//TRAJ37*02	9,982	39	106
HuT78	TRAV20-TRAJ24	NA	TRAV20*01//TRAJ24*02	13,618	55	106
HuT78	TRBV13-TRBJ1-2	Yes	TRBV13*01/TRBD1*01/TRBJ1-2*01	226,032	106	106
HuT78	TRBD2-TRBJ2-3	Yes	ND	NA	NA	106
HuT78	TRBD1-TRBJ1-2	No	ND	NA	NA	106
Pos Ctrl (RNA)	NA	NA	TRBV12-3*01//TRBJ1-2*01	46,029	9	9

*N.A., not analyzed; N.D., not detected*.

### Complexity of TRA/TRB Clonotypes in Clinical Samples

Next, we evaluated the TRA/TRB repertoire of T cells in the clinical samples. Contrary to the cell lines CD3^+^ T cells from the clinical samples showed equal TRA and TRB transcript levels (Figure 3). All V(D)J combinations of the clinical samples were analyzed and are shown in Figure 4B. Even though the exact V(D)J profiles differed between sample 1 and sample 2, their TR clonotype complexity was rather similar. To investigate if the single cell method would be capable of detecting skewed TR repertoires in clinical samples, the number of unique V(D)J combinations was determined in sample 1 (skewed TRB repertoire) and compared to sample 2 (broad TRB repertoire) (Supplementary Figure 3). This revealed that the majority of the cells of both clinical samples expressed unique V(D)J clonotypes, both for TRA and TRB. With only up to 9 identical TR clonotypes being detected in about 200 cells analyzed per cell sample, the repertoire of both clinical samples appeared diverse, probably illustrating that this number of analyzed cells in the current setting is not sufficient to distinguish skewed from broad TR repertoires.

### Association of Single Cell TRB Clonotype With Single Cell TCRVβ Staining Detected by Flow Cytometry

**Figure 5:**
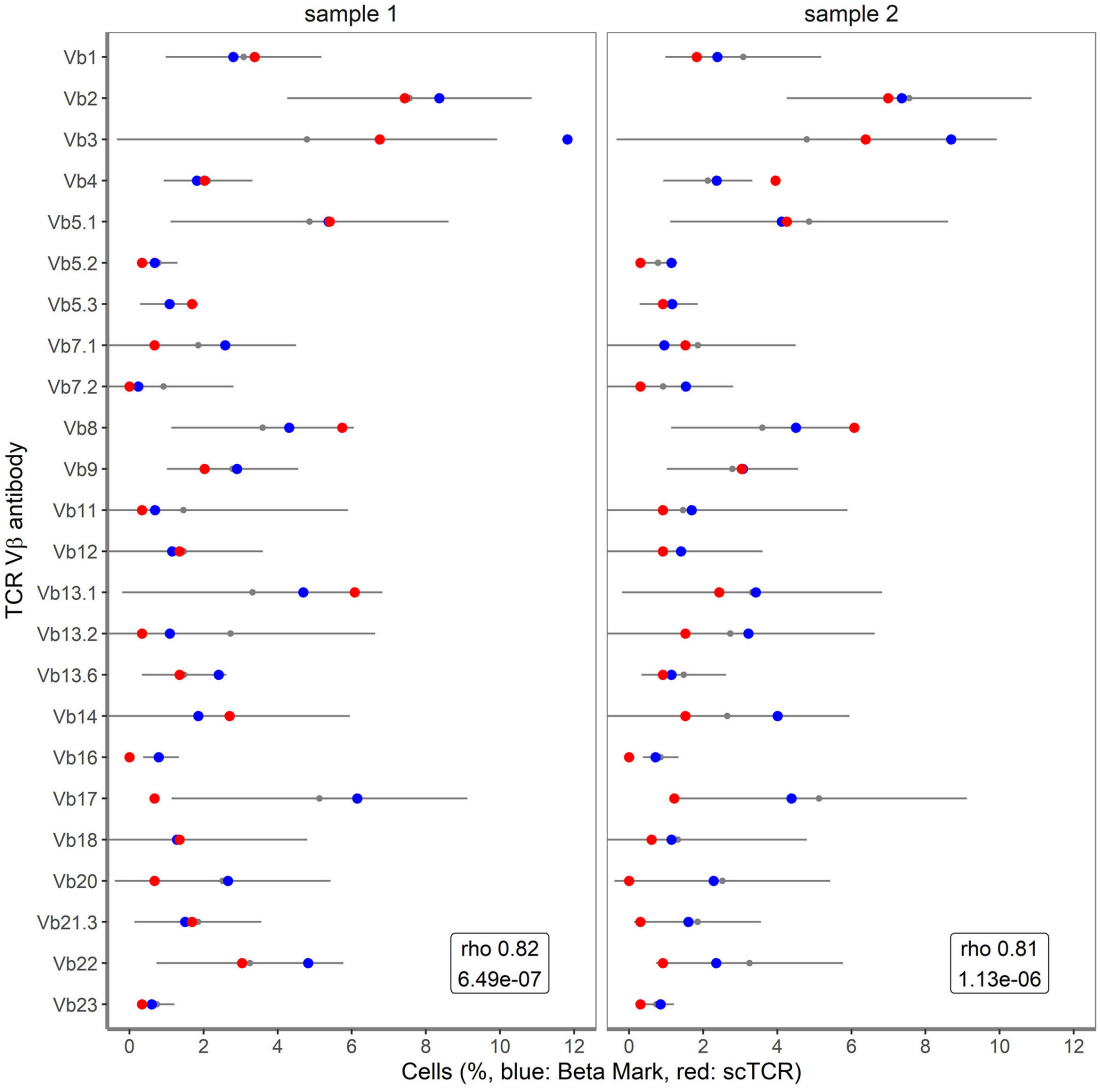
Correlation of single cell TRB clonotypes with BetaMark flow-based single cell TCRVβ test. Shown are the percentages of 24 TCRVβ families for sample 1 and sample 2 as obtained using BetaMark kit (depicted in blue) together with the TCRVβ reference values as determined using 10 healthy donors (depicted with black line) vs. single cell TRB clonotypes (depicted in red).

To further validate the accuracy of single cell TRB clonotype detection in clinical samples, we took advantage of detailed flow cytometry-based TCRVβ repertoire data for CD3^+^ T cells of the clinical samples (16). In the flow cytometry approach three different TCRVβ families were measured concurrently in 8 different tubes, with 0.5–1 million of CD3^+^ T cells per tube. The resulting profile of the 24 different TCRVβ families was compared to the TRB profile obtained by the combined single cell mRNA-based assay (Figure 5). The Spearman's Rho correlation coefficients between the datasets amounted to 0.82 for sample 1 (*P* = 6.49^*^10^−7^) and 0.81 for sample 2 (*P* = 1.13^*^10^−6^). For this comparison, sample 3 was not taken into consideration, as the viability was only 50%, resulting in an inadequate representation of TRB clonotype transcripts. Altogether, the transcript-based single cell assay showed a fairly good correlation with the protein-based single cell flow cytometry assay for TRB clonotypes.

### Cell Types Within the Clinical Samples

**Figure 6:**
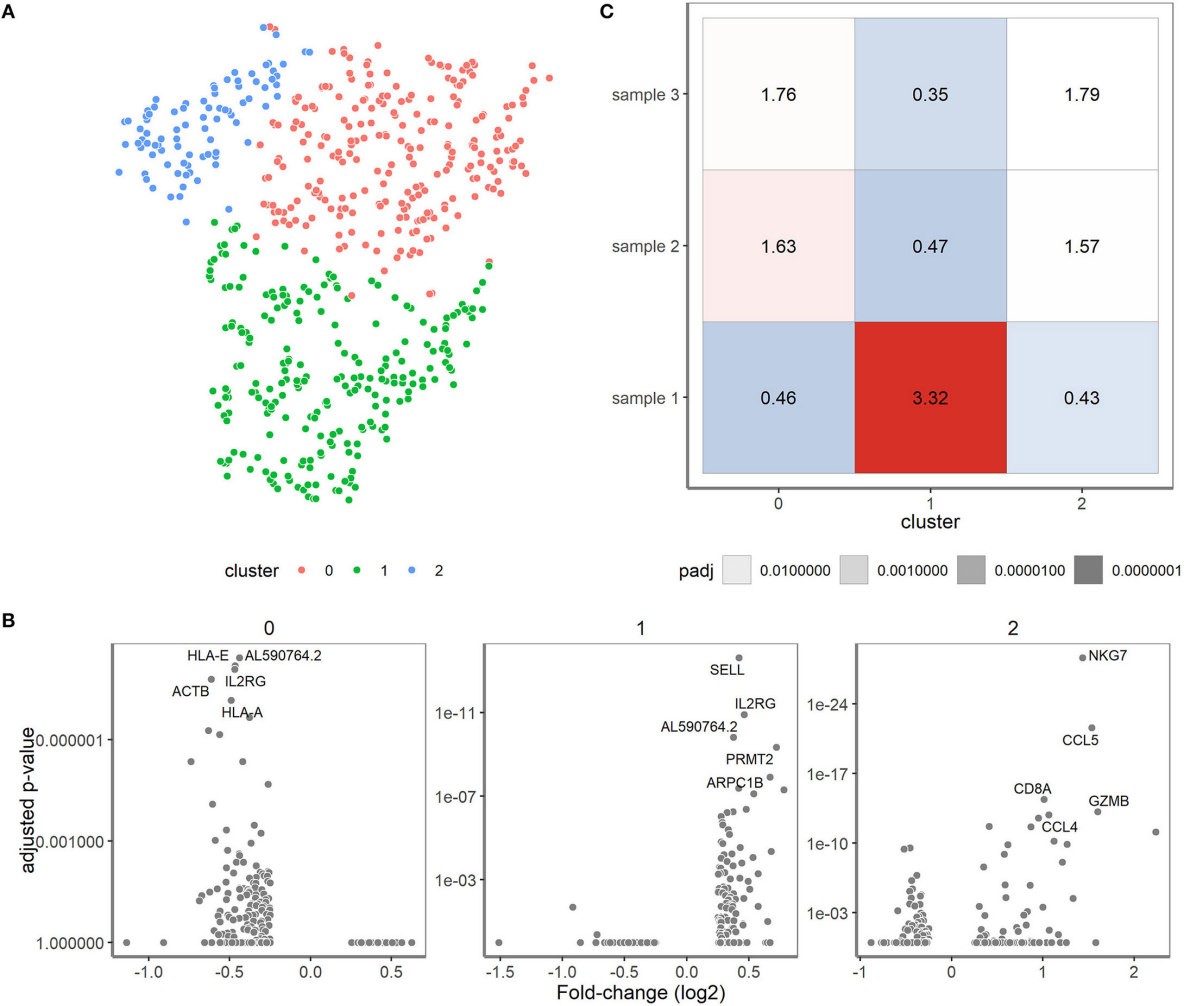
Single-cell transcriptomics reveals three cell type-specific clusters for the clinical samples. **(A)** t-SNE visualization showing the clusters. Cluster 0 represents cells mainly negative for CD4 and CD8 (depicted in red), cluster 1 predominantly consists of CD4^+^ T cells (depicted in green) and cluster 2 that of CD8^+^ T cells (depicted in blue). **(B)** Differential expression analysis of the cells in the cluster vs. the cells outside of the cluster. The fold changes (X-axis) and adjusted *P*-values (y-axis) are depicted for the genes and top 5 marker genes per cluster are shown. **(C)** Over-/underrepresentation of each of the clinical samples is depicted for the three clusters.

Finally, we zoomed in on the transcriptomes of the clinical samples and valuated whether expression differences might correlate with the skewed/clonal nature of the TRB repertoire (15) or with clinical features. In the initial *t*-SNE projection, the clinical samples were projected as a complex shape indicating the presence of different T cell types in these samples (Figure 2). We therefore clustered the cells of the different clinical samples based on their expression patterns, which resulted in three separate clusters (Figure 6A). These three clusters roughly corresponded to the presence or absence of the classical T cell markers CD4 and CD8. In cluster 1, CD4 was predominantly expressed and cluster 2 contained many cells with high CD8 expression. Cluster 0 represented single cells in which low levels of CD8 and CD4 transcripts were present (see also Figure 2). Furthermore in cluster 1, genes important for homing to secondary lymphoid organs (*SELL*) and T cell maintenance (*IL2RG*) were expressed (Figure 6B). Cluster 2 represented genes associated with cytotoxic effector function as would be expected for the predominant CD8-expressing T cell population within this cluster. In cluster 0, genes were primarily downregulated compared to the cells in clusters 1 and 2.

Over- and under-representation of cell types in the three clinical samples was subsequently determined using a Fisher exact test (Figure 6C). In general, sample 1 had a distinct transcriptome profile compared to sample 2 and 3, which were more alike (Figure 6C). Interestingly, sample 2 and 3 appeared to have more cells belonging to cluster 2 when compared to sample 1. Of note, sample 2 and 3 were both derived from cytomegalovirus (CMV)-seropositive individuals, whereas sample 1 was from a CMV-seronegative individual, which might well explain differences in the number of CD8^+^ T cells. Previous research revealed skewing to primarily occur within CD8^+^ memory T cells (15, 16), but since only 19 cells within the skewed sample 1 expressed *CD8* we could not confirm their character based on transcriptome profile.

## Discussion

In this study, we evaluated the combined assay for transcriptome and TRA/TRB repertoire on the ICELL8 Single-Cell System in view of applications to interrogate heterogeneous, paucicellular clinical samples. Our results show that it is possible to accurately study ~100–200 cells/sample with respect to parallel transcriptome and TRA/TRB clonotype repertoire analysis. Comparison of T cell lines to paucicellular clinical samples enriched for T cells revealed distinct transcriptome profiles and allowed to discriminate T cells with a more or less clonal nature (cell lines) from those possessing a complex TRA/TRB clonotype repertoire (clinical samples).

One of the first single cell approaches to link TCR-sequencing data to T cell functional phenotype was based on RT PCR and made use of primers for TCR and a limited number of T-cell related functional genes (18). Since then, single cell RNA sequencing platforms have been developed to unravel the full transcriptome in an unbiased manner (9). These allow for characterization of heterogenous and rare cell populations (10) unlike the bulk-based approaches (Table 3). Bulk NGS predominantly focuses on TRB clonotypes as analysis of TRA clonotypes is more challenging. Protein-based analysis of TCR repertoire at a single cell level using a flow cytometry-based platform also mainly focuses on the TCRVβ repertoire. The limited availability of different TCRVβ antibodies does not allow for simultaneous evaluation of TCRVβ and TCRVα families. Single cell RNA sequencing does allow for combined analysis of the TRA/TRB repertoire. The TRA/TRB repertoire can either be deduced from transcriptome data using computational methods (19) or analyzed following a TCR-specific PCR from cDNA generated from a single cell like employed by the ICELL8 Single-Cell System. Each platform has its own features making it a more or less appropriate platform to choose based on the population of interest, i.e., being a more homogeneous or heterogeneous pool of cells, starting amount of cells, etc. (9). Both 10xGenomics (20), a droplet-based platform, as well as the ICELL8 Single-Cell System, a chip-based platform, have developed a workflow for combined TCR repertoire and transcriptome analysis at the single cell level and both methods are constantly improving with respect to handling low amounts of starting material.

**Table 3:** Comparison of different platforms for studying TCRαβ repertoire diversity at the single cell level.

	**ICELL8 Single-Cell System methodology**	**10x Genomics methodology**	**NGS sequencing**	**TCRVβ profiling**
Material type	RNA-based	RNA-based	DNA/RNA-based	Protein-based
TRA/TRB combination	+	+	± (TRA is challenging)	- (TCRVα Ab panel limited)
Combined sc TRA/TRB and transcriptome	+	+	–	n.a.
Single cell approach	+	+	–	+
Range of cells/sample	50–1,800^*^	500–10,000	Range 10,000–100,000	10,000–4,000,000

*n.a., not applicable*,

* *due to Poissions distribution of single cells ~1/3 of the chip can be filled*.

Validation of the TRA/TRB clonotype composition of single cells dispensed from cell lines against bulk sequencing using Sanger and/or NGS confirmed clonotypes, indicated that low numbers of cells of a more or less homogeneous nature can be used for interrogating the TRA/TRB clonotype repertoire. Moreover, single cell analysis of TRA/TRB clonotypes showed increased resolution and revealed minor clonotypes that were missed using Sanger sequencing, albeit that these could also be found in bulk NGS analysis. Clinical samples were distinct from cell lines and revealed increased complexity with respect to their TRA/TRB repertoire. The fact that a lower frequency of cells dispensed from clinical samples yielded TRA/TRB clonotype results when compared to the cell lines, could be attributed to several factors. First, and perhaps most likely, TRA/TRB transcript levels might be lower in quiescent cells when compared with cell lines. Second, enrichment for CD3^+^ T cells was not 100%, resulting in the presence of some CD3^−^ cells amongst those dispensed, which are typically lacking TRA/TRB clonotypes. Third, ~0.5–10% of CD3^+^ T cells in the circulation are TCRγδ+ T cells (21), which would thus be lacking TRA/TRB transcripts as well.

We have previously described that at the bulk DNA level, clinical samples with a polyclonal TRB repertoire could be distinguished from those possessing a more skewed, oligoclonal TRB repertoire. In fact, detailed analysis of the TRB repertoire revealed that memory T cells, in particular CD8^+^ memory T cells of elderly patients suffering from end-stage renal disease have an oligoclonal TRB repertoire (15). In the current single cell study, we were unable to distinguish between clinical samples having a broad TRB repertoire and those having a skewed TRB repertoire at the single cell level. This might be caused by the fact that the clinical samples used in our current study, although enriched for T cells, still contained cells of a heterogeneous nature, i.e., both CD4^+^ and CD8^+^ T cells, and both antigen-inexperienced (naïve) and antigen-experienced (memory) T cells. All these cells have different phenotypes, functions and TRA/TRB repertoires. The proportion of memory T cells dispensed for the clinical samples, used in this study, might have been too low and still even too diverse with respect to the TRA/TRB repertoire to identify samples with a skewed repertoire. To be able to use the single cell TR analysis for specific research questions on TR repertoire skewing, larger cell numbers may be required to observe grouping of cells with a similar TR repertoire. Furthermore, prior purification of populations of interest might also facilitate addressing these questions.

In this respect one could think of activated or memory T cell fractions (19, 22). Despite the fact that clinical samples with a skewed TRA/TRB repertoire could not be distinguished from those possessing a broader repertoire of TRA/TRB clonotypes at the single cell level, differences in transcriptome profiles were observed between quiescent paucicellular clinical samples. These could be linked to clinical characteristics of the samples. Clinical sample 1 was distinct from sample 2 and 3, with the latter two showing more similar transcriptome profiles with increased transcripts of genes associated with cytotoxic effector function. This might be caused primarily by the fact that the latter two represent CMV-seropositive individuals, as different age categories did not distinguish these two samples from each other. CMV is known to be a major factor shaping T cell immunity, such as increasing the proportion of CD4^+^ T cells with cytotoxic features, and expanding the CD8^+^ T cell pool with increased cytotoxic potential (23–25).

Strikingly, transcripts of TRB clonotypes strongly correlated to protein levels of the 24 different TCRVβ families, indicating that TRB (and as a further implication perhaps also TRA) repertoire analysis at the single cell level is feasible in paucicellular clinical samples with a good resolution. Unfortunately we only had few samples for this comparison. Interestingly, using the flow cytometry-based assay large numbers of T cells (4–8 million) are acquired (16), compared with the 100–200 single T cells that were dispensed for TRA/TRB repertoire analysis in this study. The large cell number together with the multi-parameter nature of the flow cytometry-based approach to discriminate naïve T cells from memory T cells allows to interrogate the TCRVβ repertoire diversity of T cell subsets. However, the advantage of the current single cell approach is that a more detailed and complete TRA/TRB clonotype analysis can be performed compared with antibodies that just recognize TCRVβ domains and only cover 70% of all specificities. Furthermore, by looking at transcripts for specific markers that define naïve vs. memory subsets, single cell TRA/TRB clonotype evaluation would still be possible in the context of T cell subsets.

We noticed a higher proportion of cells from the cell lines with evaluable transcriptome data compared to clinical samples, which is most probably due to the cell lines being cultured, whereas the clinical samples were thawed and enriched for T cells, immediately before analysis. Transcript expression would be lower in such primary samples, as they are most likely not transcriptionally active. Nevertheless, transcript levels of genes specific for the cell types used in our assay, could be detected and allowed us to confirm their cellular origin. Evaluation of the transcriptome of antigen-specific T cells at the single cell level requires stimulation of clinical samples with a particular antigen. Short-term antigenic stimulation induces mRNA expression and thus offers the possibility to distinguish activated from non-activated T cells based on evaluation of specific transcripts from markers that are upregulated on the T cell membrane (26, 27). CD154 and/or CD137 are upregulated upon interaction of a specific TCR with an HLA-antigen complex on antigen presenting cells, thereby facilitating a simultaneous interrogation of the specific TRA/TRB clonotype profile of CD154- and/or CD137- expressing, activated T cells. Based on our current results, we would recommend to first enrich for these antigen-specific/activated T cells using flow cytometry-based cell sorting, prior to addressing research questions concerning differences in transcriptome profiles of antigen-specific T cells in time or between different clinical samples. Selection of antigen-specific T cells following stimulation using flow-cytometry-based cell sorting has indeed been applied by Huang et al. (22). These authors used SELECT-seq, a tool applied to specifically analyse transcriptome and TCR composition of over 3000 antigen-specific T cells by first employing a targeted PCR on Smart-Seq2-derived cDNA libraries from sorted antigen-specific CD137^+^ or CD154^+^ T cells. This allowed for identification of duplicates of TCR sequences resembling clonally expanded antigen-specific T cells, whereafter cells are selected for in-depth single cell RNA sequencing (22). Elthala et al. showed that low cell numbers, i.e., 21 cells per condition, of antigen-specific T cells could be used successfully for studying transcriptome as well as TCR repertoire, taking TCR related reads from transcriptome data using computational methods (19).

In this study, we initially started with a concentration of 0.75–2 × 10^6^ cells and a sample volume of at least 2 mL. However, only a fraction of the suspension was used to mimick working with paucicellular samples. For actual paucicellular clinical or biological samples such starting cell concentrations and volumes would often be challenging, or virtually impossible. Optimization of dispension of cells in a chip would thus be required, which could be achieved by using a different dispension method. The currently used Takara technology (13) dispenses according to the limiting dilution method, thus allowing for ~1/3 (Poisson) of the chip to be filled with single cells, while the other wells of the 72-by-72 chip contain either no cells or >1 cell and are therefore not further processed. The CellenOne dispenser (28), however, allows for a more efficient dispension of wells, thus increasing opportunities for analyzing lower volumes of clinical samples. Importantly, dispension of low numbers of cells of clinical samples in duplicate on one chip or on two different chips did not affect reproducibility, suggesting that downstream processing of a sample containing low cell numbers is not compromised. Further research should therefore focus on optimizing dispension methods to increase optimal use of cells in paucicellular clinical samples. Additionally, the minimal number of T cells required to identify samples/cells with a skewed/oligoclonal TRA/TRB repertoire should be assessed, although it should be noted that the latter would also be dependent on the nature of the samples used.

In conclusion, we showed that the ICELL8 Single-Cell System enabled combined interrogation of both the TRA/TRB repertoire and the transcriptome of paucicellular clinical samples. The single cell TR assay was further validated against the BetaMark TCRVβ single cell flow assay. This opens the way to study the response of single T cells within heterogeneous samples for both their transcriptome and TRA/TRB clonotypes in disease or upon treatment.

## Materials and Methods

### Cell Samples

In order to validate the ICELL8 Single-Cell System (Takara Bio, Shiga, Japan) for combined single cell transcriptome and TR profiling for clinically relevant samples, we used two different cell lines, with known TRA/TRB clonotypes (14) as deduced from Sanger as well as NGS analysis data (Table 1). In addition, we used three different clinical samples, one of which had a skewed/oligoclonal TRB repertoire (Table 1, sample 1) and two of which showed a broad/polyclonal TRB repertoire (Table 1, sample 2 and sample 3), as determined by DNA-based TRB spectratyping (15). Clinical samples had been immunophenotyped in depth with respect to the presence of the different T cell subsets as well as their differentiation status (29, 30). PBMCs from these samples had been isolated from heparinized blood using Ficoll-Paque Plus (GE Healthcare, Uppsala, Sweden) density centrifugation (31), and stored in liquid nitrogen with a minimum amount of 10 × 10^6^ cells per vial. In addition to TRB spectratyping on bulk DNA (15), a detailed characterization of the TCRVβ repertoire for the different T cell populations had been performed previously using the TCRVβ antibody kit (IOTest® Beta Mark, Beckman Coulter Nederland B.V., Woerden, Netherlands) (16). For sample 1 (skewed TRB repertoire), two TCRVβ families were more prevalent compared to reference values, i.e., TCRVβ3 and TCRVβ13.6, corresponding to TRB28 and TRB6-6 in the IMGT nomenclature, respectively. To validate the combined single cell transcriptome and TRA/TRB profiling assay for these clinical samples, 1 vial of ~10 million PBMCs was thawed and cell viability was assessed using trypan blue (Supplementary Table 1). Two out of three samples contained <20% of dead cells whereas the third one contained 50% of dead cells. Samples were enriched for T cells, in an untouched manner, using the PAN T cell isolation kit (Miltenyi Biotec B.V., Bergisch Gladbach, Germany) according to the manufacturer's instructions. Following this enrichment step, the frequency of CD3^+^ T cells amounted to >90%. Sorted cell samples were resuspended in degassed sterile PBS (without Mg^2+^ and Ca^2+^, Invitrogen; Landsmeer, Netherlands), pH 7.4 at room temperature at a concentration 2 × 10^6^/mL. To mimick analysis of samples with low cell numbers, from these cell suspensions eventually 100–200 cells were dispensed in the chip (see below).

### Combined Single Cell Transcriptome and TRA/TRB Repertoire Analysis

#### Cell Dispension

Cells were stained with Hoechst and propidium iodide (PI) using the Ready Probes Cell viability Imaging kit (Thermo Fisher Scientific, Waltham (MA), USA) according to the manufacturer's recommendation. Cells were dispensed into nanowells of a blanco chip using the ICELL8 Single-Cell System (Takara Bio) to determine the optimal cell concentration yielding a Poisson distribution value between 0.8 and 1.2 for optimal single cell dispension. Based on the obtained Poisson distribution value, cells were diluted and dispensed in a pre-printed chip with the ICELL8 Single-Cell System, using a suspension of 20 K cells/μl. Each pre-printed TCR chip contains 1,728 unique barcodes and each barcode is pre-printed three times on the chip (Takara Bio). Positive control nanowells were dispensed with 5 pg Jurkat RNA and negative control nanowells with 1x PBS. After dispensing cells, the chip was imaged with a 4x objective for Hoechst and PI fluorescent signal and analyzed using CellSelect automated microscopy image analysis software (Takara Bio). Wells with positive Hoechst signal and negative for PI signal were selected for further processing, whereasempty wells and wells with multiple single cells were excluded.

#### cDNA Synthesis

Fifty nanoliter of SMART RT-PCR reagents (Takara Bio) were dispensed to all selected wells and full-length cDNA was amplified in-chip for 24 cycles using SMARTScribe reverse transcriptase according to manufacturer's instructions (Figure 1B). Barcoded amplicons were collected into a tube by centrifugation and off-chip purified with the NucleoSpin Gel and PCR Clean-up kit (Machery-Nagel, Düren, Germany) followed by an AMPure XP beads purification (Beckman Coulter, Brea, USA). cDNA synthesis, purification and size selection were checked on a Bioanalyzer using the High sensitivity DNA kit [Agilent Technologies, Santa Clara (CA), USA]. The resulting cDNA library was split for either TRA/TRB amplification or whole transcriptome amplification.

#### TR Amplification

TRA and TRB transcripts were amplified by PCR for 16 cycles using primers specific for human TRA and TRB constant region sequences, respectively, according to the manufacturer's instructions (Figure 1B). A semi-nested PCR was performed for 14 cycles to further amplify transcripts from both TR loci and to add the Illumina adaptor sequences (Figure 1B). The resulting TR sequencing libraries were purified and size-selected for ~400–900 bp fragments with AMPure XP beads. Sequencing libraries were quantified by Quant-it assay, diluted, and checked on a Bioanalyzer using the High sensitivity DNA kit. From the sequencing libraries, paired-end reads of 300 bp length were sequenced with an 8 bp index on an Illumina MiSeq sequencer according to the protocol by Illumina [San Diego (CA), USA].

#### 5′-Specific WTA

For transcriptome analysis, part of the purified cDNA was tagmented using the Nextera XT DNA library prep (Illumina) and Illumina adaptor and indices sequences were extended by a 12 cycle PCR according to the manufacturer's instructions (Figure 1B). The resulting whole transcriptome sequencing libraries were purified and size-selected for ~400–900 bp fragments with AMPure XP beads. The sequencing libraries were quantified with the Quant-it assay and checked on a Bioanalyzer using the High sensitivity DNA kit. Sequencing libraries were sequenced according to the Illumina TruSeq Rapid v2 protocol on Illumina HiSeq2500 sequencer. Single reads were generated of 50 bp in length with a single 8 bp index sequence.

#### Transcriptome Analysis

For RNA expression data analysis well-barcodes and Illumina adapter sequences were removed from the reads using in-house developed software. The remaining sequences were aligned to the GRCh38 human reference genome extended with exon-exon junctions using the HISAT2 aligner (32). Resulting alignments were annotated with the well-barcodes and converted to BAM format using SAMtools (33). The alignments were converted to BED entries and intersected using Ensembl exons (release 96) (34) using BEDtools (35). Per gene quantification was then performed with further in-house software.

Subsequent analysis was performed using the Seurat single cell analysis package version v3.1.4 (36) in R (version 3.6.3). Cells were filtered from the analysis, if a cell had more than 5% mitochondrial signal, fewer than 500 or more than 5,000 genes, the latter based on the assumption that too many detected genes are most likely the result from doublets of cells instead of single cells (36). The expression signals were scaled and normalized using the appropriate methods from Seurat and dimensional reduction was performed using a principal component analysis based on the 2,000 most highly variable (protein coding) genes. The first 5 dimensions in the dataset were used for the *t*-SNE visualization and cell-type analysis.

In the cell type analysis, cells were first clustered based on their expression profiles (top 5 dimensions, resolution 0.4). To gain insights into the cell type represented by the clusters, differential expression analyses were performed of the clusters compared the other cells. Using the Fisher exact method, the over- and under-representation of samples in the clusters were tested.

#### TR Analysis

Forward and reverse reads for the TR data were merged using PEAR (version 0.9.2) (37). The assembled sequences were then aligned to IMGT-defined TR V, D, and J reference sequences (38) using the IgBLAST tool (version 1.14) (39). The resulting alignments were processed further in R (version 3.6.3). Throughout the analysis, the tidyverse packages for R were used (40).

## Data Availability Statement

The datasets presented in this study can be found in the online NCBI repository at: https://www.ncbi.nlm.nih.gov and accession number PRJNA630589.

## Ethics Statement

The studies involving human participants were reviewed and approved by Medical Ethical Committee of Erasmus MC, University Medical Center (MEC-2012-022). The patients/participants provided their written informed consent to participate in this study.

## Author Contributions

NL, AWL, MGHB, RB, and WV participated in research design, data collection, data analysis, and writing of the manuscript. ACJL and MK participated in research design and data collection. MB participated in research design, data collection, and data analysis. ZA and AD participated in data collection and data analysis. All authors contributed to the article and approved the submitted version.

## Conflict of Interest

The authors declare that the research was conducted in the absence of any commercial or financial relationships that could be construed as a potential conflict of interest.

## Supplementary Material

Supplementary Figure 1 Summary of transcriptome and TR sequencing output of different chips. Occupancy and success rate of dispensed single cells **(left)**, TR **(middle)**, and transcriptome profiles (right) visualized as successful wells (black) or unsuccessful wells (red).

Supplementary Figure 2 Overall transcript levels of clinical samples and cell lines. The number of genes (Y-axis) is plotted against the number of reads in thousands (X-axis) for each individual single cell. Individual samples are indicated at the top with their sample names.

Supplementary Figure 3 Unique TRA and TRB combinations. The number of cells with identical for TRA (**top**, blue) or TRB (**bottom**, red) clonotypes for clincal samples 1 **(left)** and 2 **(right)** is plotted against the V(D)J combination ranked for occurrence.

Supplementary Table 1 In depth characteristics of the single cell transcriptome and TCR experiments.
