# Supplementary figures and images for: Validation of a Combined Transcriptome and T Cell Receptor Alpha/Beta (TRA/TRB) Repertoire Assay at the Single Cell Level for Paucicellular Samples
Source: Front Immunol. 2020 Aug 28;11:1999. doi: 10.3389/fimmu.2020.01999 (PMC7500136; doi:10.3389/fimmu.2020.01999)

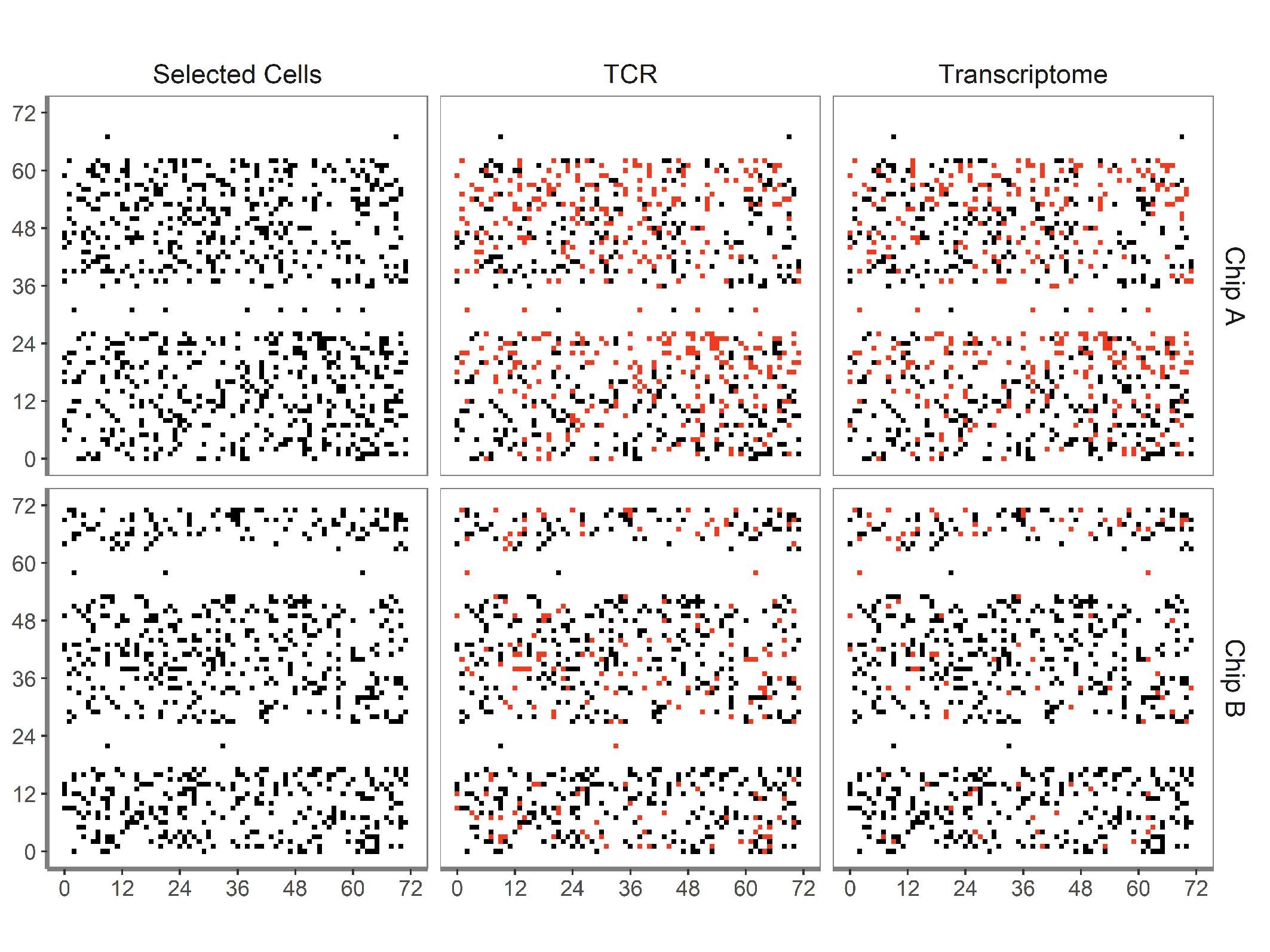

Supplement: Supplementary Figure 1 — Summary of transcriptome and TR sequencing output of different chips. Occupancy and success rate of dispensed single cells (left), TR (middle), and transcriptome profiles (right) visualized as successful wells (black) or unsuccessful wells (red). [file Image_1.JPEG]

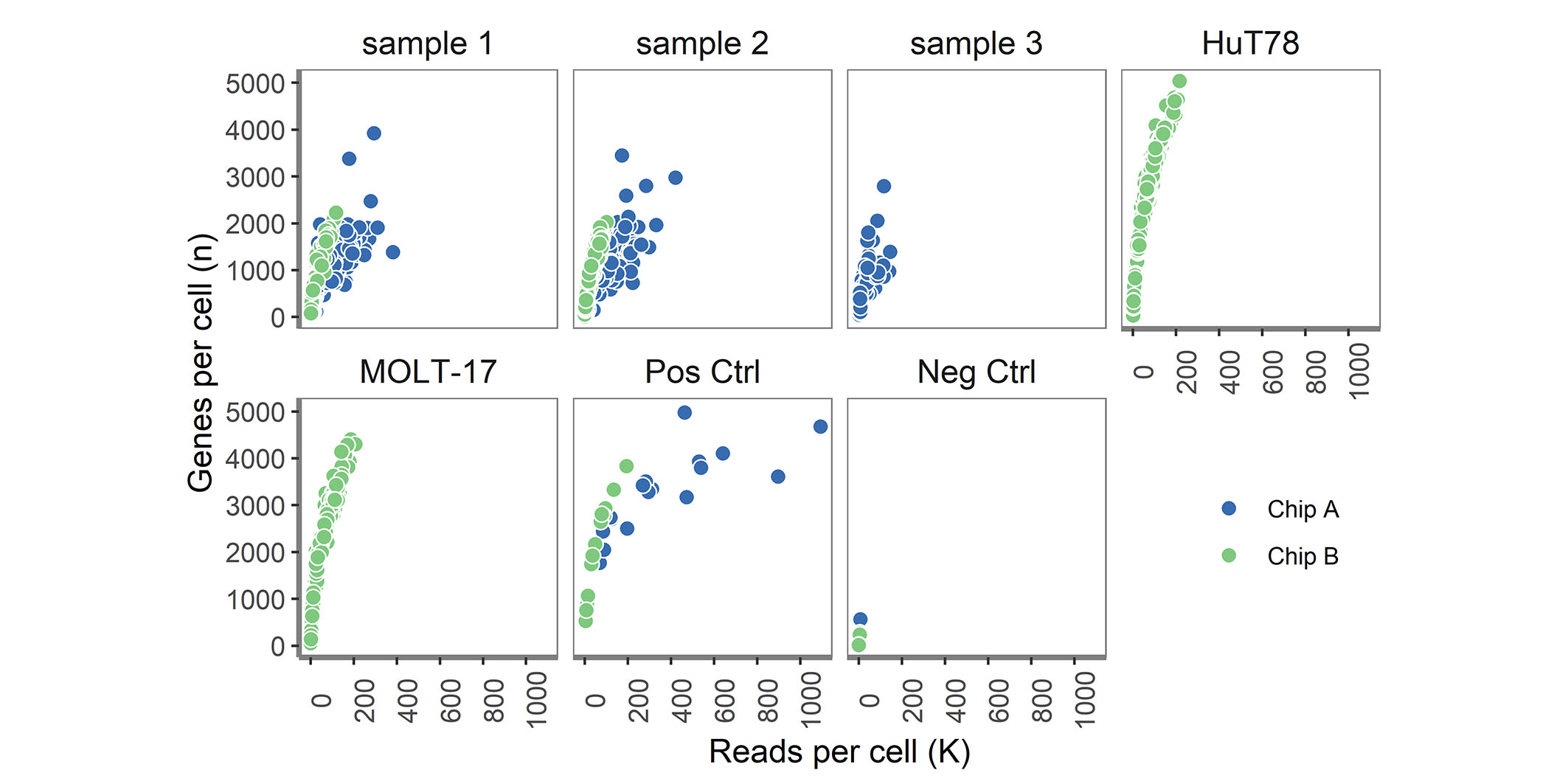

Supplement: Supplementary Figure 2 — Overall transcript levels of clinical samples and cell lines. The number of genes (Y-axis) is plotted against the number of reads in thousands (X-axis) for each individual single cell. Individual samples are indicated at the top with their sample names. [file Image_2.JPEG]

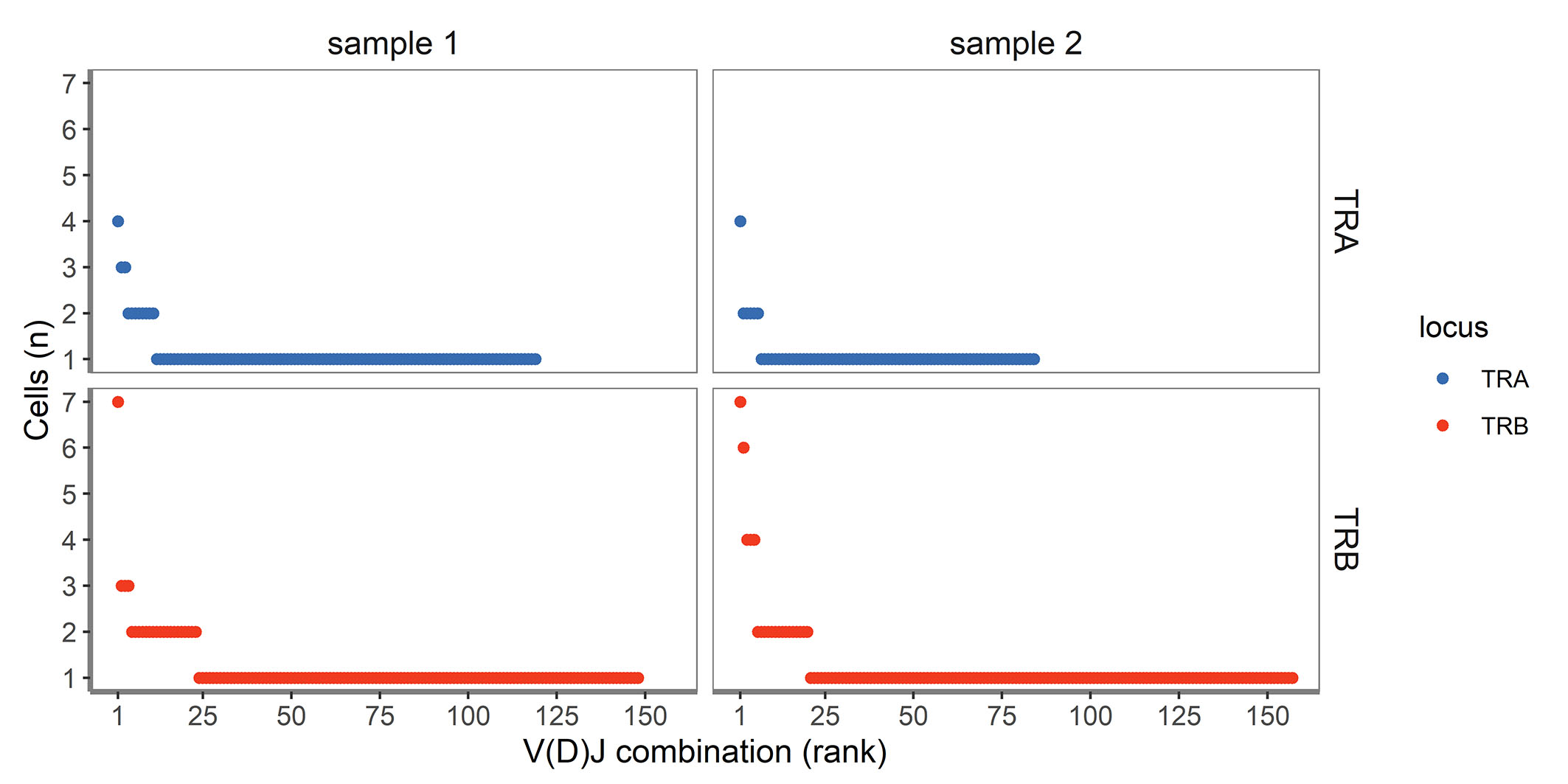

Supplement: Supplementary Figure 3 — Unique TRA and TRB combinations. The number of cells with identical for TRA (top, blue) or TRB (bottom, red) clonotypes for clincal samples 1 (left) and 2 (right) is plotted against the V(D)J combination ranked for occurrence. [file Image_3.JPEG]
